# The Potential of Salivary Biomarkers in Early Detection of Pancreatic Ductal Adenocarcinoma: A Systematic Review

**DOI:** 10.7759/cureus.55003

**Published:** 2024-02-26

**Authors:** Hamza Al Balushi, Purnashree Chowdhury, Hisham M Babu, Abdur Rehman, Syed Faqeer Hussain Bokhari, Lina M Al-Tarawneh, Abedallah J Al-Adwan, Meher Cheran, Srikar P Chilla, Anirudh R Addula, Maaz Amir

**Affiliations:** 1 Internal Medicine, First Bethune Hospital, Muscat, OMN; 2 Neurology, Comilla Medical College Hospital, Chittagong, BGD; 3 Internal Medicine, Jagadguru Sri Shivarathreeshwara (JSS) Medical College and Hospital, Mysore, IND; 4 Surgery, Mayo Hospital, Lahore, PAK; 5 Surgery, King Edward Medical University, Lahore, PAK; 6 Internal Medicine, Jordan University Hospital, Amman, JOR; 7 Internal Medicine, Jordanian Royal Medical Services, Amman, JOR; 8 Internal Medicine, American International Medical University, Gros Islet, LCA; 9 Medicine, Care Hospitals, Hyderabad, IND; 10 School of Health Sciences, University of East London, London, GBR; 11 Internal Medicine, King Edward Medical University, Lahore, PAK

**Keywords:** pancreatic cancer, surveillance, review, pancreas, saliva, early detection, salivary biomarkers, pancreatic ductal adenocarcinoma

## Abstract

Pancreatic ductal adenocarcinoma (PDAC) is a formidable global health concern with a dire prognosis, highlighting the critical need for early detection strategies. This systematic review delves into the potential of salivary biomarkers as a non-invasive means for identifying PDAC at its incipient stages. Saliva's proximity to the circulatory system enables the detection of tumor-derived biomolecules, making it an ideal candidate for mass screening. The analysis of three selected studies reveals promising candidates such as Neisseria mucosa, Fusobacterium periodonticum, polyamines, and specific long non-coding RNAs (lncRNAs). Notably, polyamines like spermine show potential in distinguishing PDAC, while lncRNAs HOX transcript antisense RNA (HOTAIR) and plasmacytoma variant translocation 1 (PVT1) exhibit superior sensitivity and specificity compared to traditional serum markers. However, challenges, including small sample sizes and a lack of validation, underscore the need for standardized diagnostic panels and large-scale collaborative studies. Advancements in nanotechnology, machine learning, and ethical considerations are crucial for harnessing the diagnostic potential of saliva. The review emphasizes the imperative for extensive clinical trials to validate salivary biomarkers, ensuring not only diagnostic accuracy but also cost-effectiveness, patient compliance, and long-term benefits in the realm of PDAC screening. Longitudinal studies are recommended to unravel temporal changes in salivary biomarkers, shedding light on disease progression and treatment response.

## Introduction and background

Pancreatic ductal adenocarcinoma (PDAC) is projected to become the second leading cause of cancer-related deaths by 2030. It has an overall five-year survival rate of merely 8%, which drops to only 3% for metastatic disease [1]. Most patients are diagnosed at an advanced stage when curative surgery is no longer feasible [2]. Hence, there is a crucial unmet need for developing effective modalities for early PDAC detection. Saliva has emerged as an ideal diagnostic biofluid for non-invasive sampling. The proximity of salivary glands to the circulatory system allows tumor-derived biomolecules to be readily detectable in saliva [3]. Saliva collection is easy, non-invasive, and cost-effective compared to drawing blood. It is also more convenient for mass screening compared to imaging techniques [4]. Over the past decade, the applicability of salivary diagnostics has expanded from oral diseases to various systemic conditions, including lung, breast, and pancreatic cancer [5-7]. This underscores the exciting potential of salivary biomarkers for improving PDAC outcomes through early detection.

Numerous salivary biomarkers indicative of PDAC presence have been reported, ranging from metabolites and proteins to circulating tumor DNA/RNA species, microbes, and extracellular vesicles (EVs) [5]. Alterations in metabolite levels act as red flags for malignant transformation-induced metabolic shifts. Certain metabolites, like polyamines, show elevated salivary levels due to dysregulation of key oncogenic pathways in PDAC [8,9]. Signature protein changes also arise from PDAC onset and progression. Non-coding RNA dysregulation is integral to PDAC tumorigenesis. MicroRNAs extracted from salivary EVs could effectively distinguish resectable PDAC, with significant upregulation noted in certain miRNA species [10]. The oral microbiome also exhibits characteristic changes in microbial composition and diversity during PDAC development [11].

While several promising candidate biomarkers have been discovered, most studies lack sufficient sample sizes and independent validation. No study has yet evaluated different salivary biomarker types together in a common PDAC cohort. Multi-analyte assays can greatly improve predictive ability over single biomarkers. This systematic review aims to summarize the results of studies done so far to identify the diagnostic role of salivary biomarkers in the early diagnosis of PDAC.

Hence, developing standardized salivary diagnostic panels through large collaborative studies is the logical next step to establishing the credibility of salivary testing for reliable PDAC screening and surveillance.

## Review

Materials and methods

Search Strategy

In the context of investigating the role of salivary biomarkers in the early detection of pancreatic ductal adenocarcinoma (PDAC), our search strategy adhered to the PRISMA (Preferred Reporting Items for Systematic Reviews and Meta-Analyses) guidelines. The systematic exploration of relevant literature was conducted across esteemed databases known for comprehensive medical and scientific reports, namely PubMed, Embase, Web of Science, and Scopus. These databases were chosen for their extensive repositories of peer-reviewed articles, ensuring a solid foundation for our systematic review of the role of salivary biomarkers in the early detection of PDAC.

The search was guided by a carefully selected set of keywords and phrases pertinent to the study objectives, including terms such as "Salivary Biomarkers," "Pancreatic Ductal Adenocarcinoma," "Early Detection," and related terms. Boolean operators "AND" and "OR" were strategically employed to create a comprehensive search algorithm. For instance, the string "Salivary Biomarkers AND Pancreatic Ductal Adenocarcinoma AND Early Detection" was utilized to focus on studies explicitly examining the early detection of PDAC through salivary biomarkers. The use of "OR" facilitated the inclusion of broader terms associated with salivary biomarkers and PDAC, ensuring a thorough exploration of the literature.

To maintain a contemporary and relevant scope, our search was restricted to studies published from the inception of each database until December 2023. This timeframe allowed for the inclusion of historical and cutting-edge research, offering a comprehensive overview of the role of salivary biomarkers in the early detection of PDAC. Filters were applied to include studies in the English language and involving human subjects, aligning with the specific objectives of our review.

Additionally, manual searches of the reference lists of included studies and relevant reviews were conducted to ensure a thorough exploration of the literature. This rigorous and expansive search strategy aimed to capture the full spectrum of evidence concerning the role of salivary biomarkers in the early detection of PDAC, providing a robust foundation for our systematic review.

Eligibility Criteria

In establishing eligibility criteria for our systematic review of the role of salivary biomarkers in the early detection of PDAC, precision and relevance were paramount. Peer-reviewed research articles, observational studies, and clinical trials constituted the types of studies included, reflecting a commitment to high-quality, evidence-based knowledge. Our review encompassed studies investigating the utility of salivary biomarkers for early detection, with outcomes such as sensitivity, specificity, and diagnostic accuracy considered. Conversely, exclusion criteria were carefully tailored to maintain the focus and methodological rigor of the review. Studies not directly addressing the relationship between salivary biomarkers and early detection of PDAC, as well as those lacking relevant outcome measures, were omitted. Non-English-language publications, unpublished works, and gray literature, such as conference abstracts, were also excluded. Furthermore, studies involving animal models or presenting insufficient data on the role of salivary biomarkers in the early detection of PDAC were excluded, aligning with the review's commitment to human-centered, data-rich research.

Data Extraction

The data extraction process for our systematic review of the role of salivary biomarkers in the early detection of PDAC was meticulously structured to ensure the integrity of our research findings. This critical process unfolded in two stages, emphasizing thoroughness and accuracy.

In the initial stage, a preliminary screening was conducted to filter articles based on relevance indicated by titles and abstracts. Two independent reviewers performed this assessment, classifying each article as relevant, not relevant, or probably relevant based on a conscientious appraisal of the study's abstract and its pertinence to the review's focus. Progressing to the second stage, full-text articles deemed relevant or probably relevant underwent a detailed examination. Two independent reviewers utilized a standardized data extraction template within Microsoft Excel (Microsoft® Corp., Redmond, WA) to capture and organize critical information. This template provided a uniform platform for collecting data on the author(s), year of publication, study's country of origin, participant demographics, study setting, design, methodologies, salivary biomarkers investigated, outcome measures, and key findings. Both reviewers independently applied pre-established inclusion and exclusion criteria to each study, with any discrepancies resolved through the adjudication of a third independent reviewer. This meticulous data extraction process ensured that all relevant data points were captured, contributing to the robustness and credibility of our systematic review's conclusions.

Results

Study Selection Process

The study selection process for our systematic review on the role of salivary biomarkers in the early detection of PDAC adhered to the PRISMA guidelines, ensuring a transparent and systematic approach. A comprehensive search across databases initially yielded 57 records, from which nine duplicates were removed, resulting in a refined pool of 48 unique studies. Subsequent examination of titles and abstracts led to the exclusion of 43 records that did not meet predefined relevance criteria. A rigorous evaluation of the remaining five articles, involving retrieval and scrutiny of full texts, led to the exclusion of an additional two reports that did not align with stringent inclusion criteria.

**Figure 1:**
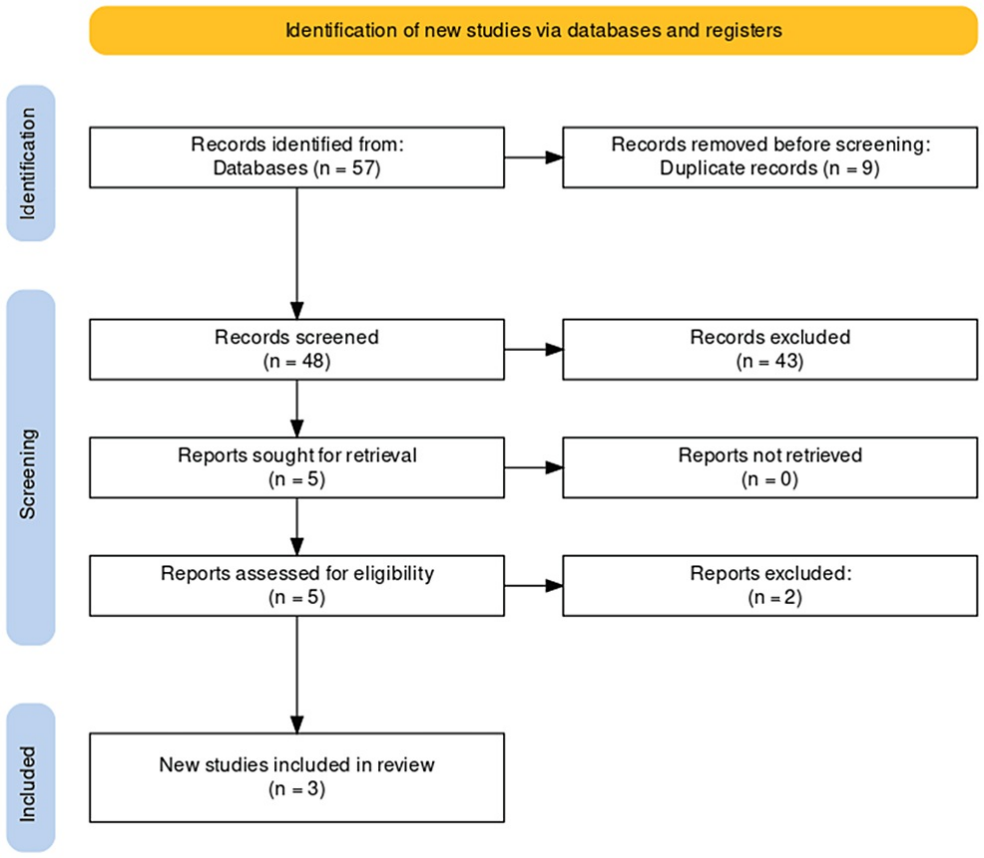
PRISMA flow diagram of selection of studies for inclusion in the systematic review.

The culmination of this meticulous selection process identified three studies deemed suitable for inclusion in our review, providing a focused and rich source of evidence for the analysis of the role of salivary biomarkers in the early detection of PDAC. The PRISMA flowchart detailing the study selection process is presented in the following figure (Figure 1).

Characteristics of Selected Studies

The selected studies exhibited variability in their design and scope, mirroring the multifaceted nature of salivary biomarker research in the context of PDAC. The studies encompassed a thorough examination of the impact of salivary biomarkers on early detection outcomes, including sensitivity, specificity, and diagnostic accuracy. Furthermore, these studies explored the interplay between salivary biomarkers and various diagnostic modalities relevant to early PDAC detection. The diversity in study design and methodology facilitated a comprehensive cross-sectional view of the current understanding of how salivary biomarkers contribute to the early detection of PDAC. This rich diversity of perspectives significantly enhanced the synthesis and review phases of our systematic review.

Table 1 outlines the key details of each selected study, providing a comprehensive overview of their respective methodologies, salivary biomarkers investigated, and critical findings. This tabular presentation ensures transparency and facilitates a detailed examination of the individual contributions of each study to our systematic review, reinforcing the robustness of our review and synthesis of the role of salivary biomarkers in the early detection of PDAC (Table 1).

**Table 1:** A summary of the studies included in this systematic review. PC: pancreatic cancer, MLR: multiple logistic regression, AUC: area under the ROC curve, PDAC: pancreatic ductal adenocarcinoma, lncRNAs: long non-coding RNAs, HOTAIR: HOX transcript antisense RNA, HOTTIP: HOXA distal transcript antisense RNA, MALAT1: metastasis-associated lung adenocarcinoma transcript 1, PVT1: plasmacytoma variant translocation 1.

Author	Year	Study type	Biomarker studied	Main findings
Sun et al. [12]	2020	Cross-sectional	Neisseria mucosa, *Fusobacterium periodonticum*	*Neisseria mucosa* was identified as a potential protective biomarker, while *F. periodonticum* was associated with PC. Functional predictions indicated altered pathways related to glucose metabolism and inflammation in PC.
Asai et al. [9]	2018	Cross-sectional	Polyamines	An MLR model, including alanine, N1-acetylspermidine, 2-oxobutyrate, and 2-hydroxybutyrate, showed high discrimination ability with an AUC of 0.887. Polyamines, especially spermine, exhibited significant differences between control and PDAC. The combination of salivary metabolites demonstrated potential for detecting PDAC, providing a promising avenue for further research and validation with larger cohorts.
Xie et al. [13]	2016	Cross-sectional	lncRNAs: H19, HOTAIR, HOTTIP, MALAT1, PVT1	HOTAIR and PVT1 showed elevated levels in PC tissues and saliva compared to healthy controls and benign tumors. Their discriminatory power for PC detection was evaluated, demonstrating good sensitivity and specificity, outperforming serum CA19-9. Salivary levels decreased after curative pancreatectomy. The findings suggest HOTAIR and PVT1 as potential non-invasive biomarkers for PC detection.

Quality Assessment

The quality assessment of the three selected cross-sectional studies for this systematic review was conducted using the Newcastle-Ottawa Scale (NOS), a widely accepted tool for evaluating the methodological quality of cross-sectional studies (Table 2). Two studies were of fair quality, and one study was of good quality based on the assessment results.

**Table 2:** Quality assessment of the included studies using the Newcastle-Ottawa quality assessment scale.

Authors	Selection	Comparability	Outcome	Overall quality rating
Sun et al. [12]	★★★☆	☆☆	★☆☆	Fair
Asai et al. [9]	★★☆☆	★★	★☆☆	Fair
Xie et al. [13]	★★☆☆	★★	★★★	Good

Discussion

This systematic review offers a comprehensive overview of the current knowledge of salivary biomarkers for PDAC detection. We evaluated studies assessing various PDAC saliva biomarkers, including metabolites, proteomic markers, circulating tumor DNA/RNA, microbes, and EVs. Notably, polyamines like spermine and N1-acetylspermidine showed significantly elevated levels in PDAC patient saliva, indicative of cellular polyamine dyshomeostasis associated with PDAC oncogenesis [8,14]. Circulating tumor RNA species, such as miR-3679-5p and the long non-coding RNA PVT1, were differentially expressed in PDAC saliva compared to controls, highlighting their regulatory function and diagnostic value [10,15]. Global shifts in oral microbial composition and diversity during PC development reflect tumor-induced systemic effects [16]. The results of the included studies suggest that combining multiple biomarker types can drastically improve predictive performance compared to individual markers. Salivary EV miRNA panels, in particular, demonstrated high classification accuracy for PDAC diagnosis [14].

The field of salivary diagnostics has advanced significantly, particularly with nanotechnology-based sensors and microfluidic devices. These technologies enhance the sensitivity and specificity of biomarker detection and enable the analysis of a broad spectrum of biomarkers, from nucleic acids to proteins and metabolites. While saliva offers several advantages over other biofluids, such as non-invasiveness and ease of collection, it also presents unique challenges. The lower concentrations of biomarkers in saliva necessitate highly sensitive detection technologies. Additionally, saliva composition can be influenced by various factors, including dietary habits, oral health, and systemic diseases, potentially impacting the reliability of biomarker measurements [17]. Addressing these challenges is essential to fully harnessing saliva's potential for PDAC diagnostics.

The integration of artificial intelligence and machine learning in salivary diagnostics represents a paradigm shift. These technologies can effectively analyze complex biomarker profiles, identify diagnostic patterns, and predict disease progression. Machine learning algorithms can handle the variability and multifactorial nature of salivary biomarkers, enhancing the accuracy of PDAC detection and enabling personalized diagnostic approaches [18]. One of the critical challenges in salivary diagnostics is the standardization of sample collection, processing, and analysis. Variability in these procedures can lead to inconsistent results, which is a significant barrier to clinical implementation. The use of salivary diagnostics, especially those involving genetic and epigenetic markers, raises ethical and privacy concerns. Ensuring patient consent, safeguarding genetic information, and addressing potential discrimination based on genetic risk factors are vital aspects that need consideration.

To translate the potential of salivary biomarkers into clinical practice, extensive research and clinical trials are necessary. These trials should focus not only on diagnostic accuracy but also on cost-effectiveness, patient compliance, and the long-term benefits of saliva-based screening for PDAC. Moreover, longitudinal studies could provide insights into the temporal changes in salivary biomarkers, aiding in understanding disease progression and response to treatment [17].

## Conclusions

Salivary biomarkers hold promise for early detection of PDAC, offering a non-invasive and convenient screening method. The reviewed studies underscore the potential of specific biomarkers, including polyamines and lncRNAs, but highlight the need for larger, validated studies and standardized diagnostic panels. Advancements in nanotechnology and machine learning, coupled with addressing ethical considerations, are essential for realizing saliva's diagnostic potential. Extensive clinical trials are imperative for evaluating the cost-effectiveness, patient compliance, and long-term benefits of saliva-based PDAC screening, emphasizing the necessity of a comprehensive and collaborative approach to advancing this field.
